# Evaluating the transferability of Hapmap SNPs to a Singapore Chinese population

**DOI:** 10.1186/1471-2156-11-36

**Published:** 2010-05-07

**Authors:** Anand Kumar Andiappan, Ramani Anantharaman, Pallavi Parate Nilkanth, De Yun Wang, Fook Tim Chew

**Affiliations:** 1 Department of Biological Sciences, National University of Singapore, Science Drive 4, Singapore 117543; 2 Department of Otolaryngology, National University of Singapore, 10 Lower Kent Ridge Road, Singapore 119260

## Abstract

**Background:**

The International Hapmap project serves as a valuable resource for human genome variation data, however its applicability to other populations has yet to be exhaustively investigated. In this paper, we use high density genotyping chips and resequencing strategies to compare the Singapore Chinese population with the Hapmap populations. First we compared 1028 and 114 unrelated Singapore Chinese samples genotyped using the Illumina Human Hapmap 550 k chip and Affymetrix 500 k array respectively against the 270 samples from Hapmap. Secondly, data from 20 candidate genes on 5q31-33 resequenced for an asthma candidate gene based study was also used for the analysis.

**Results:**

A total of 237 SNPs were identified through resequencing of which only 95 SNPs (40%) were in Hapmap; however an additional 56 SNPs (24%) were not genotyped directly but had a proxy SNP in the Hapmap. At the genome-wide level, Singapore Chinese were highly correlated with Hapmap Han Chinese with correlation of 0.954 and 0.947 for the Illumina and Affymetrix platforms respectively with deviant SNPs randomly distributed within and across all chromosomes.

**Conclusions:**

The high correlation between our population and Hapmap Han Chinese reaffirms the applicability of Hapmap based genome-wide chips for GWA studies. There is a clear population signature for the Singapore Chinese samples and they predominantly resemble the southern Han Chinese population; however when new migrants particularly those with northern Han Chinese background were included, population stratification issues may arise. Future studies needs to address population stratification within the sample collection while designing and interpreting GWAS in the Chinese population.

## Background

The International Hapmap Project is a multi-centre effort aimed at identifying genetic variations across the human genome among different individuals to aid biomedical researchers in identifying genetic links to various diseases and variable drug response [1-3]. The Hapmap Consortium developed a human haplotype map by genotyping 270 samples from four populations with diverse geographic ancestry. These samples included 30 trios (mother, father, and adult child) from the Yoruba in Ibadan, Nigeria (YRI); 30 trios from the Centre d'Etude du Polymorphisme Humain (CEPH) collection of Utah residents of Northern and Western European ancestry; 45 unrelated Han Chinese in Beijing (CHB); and 45 unrelated Japanese in Tokyo (JPT) [4]. While the latest published update to the Hapmap project indicates the availability of data for more than 3.1 million single nucleotide polymorphisms (SNPs) in the four populations [3] this number has grown to more than 26 million SNPs in 11 populations(NCBI). The common patterns of DNA sequence variants, their frequencies and correlations have been made available online at the Hapmap database [5] and dbSNP [6]. While the genotyping data from the four main Hapmap populations does serve as a valuable resource for linkage disequilibrium (LD) based marker selection in genetic association studies [2,7], there is a need to evaluate its extensibility to other populations. Studies comparing LD patterns and transferability of tag SNPs [8-13] have shown that allele and haplotype frequencies of independent populations are relatively similar with those obtained from the Hapmap populations. The concordance is however, not always near 100%. In analyzing regions spanning 750 kb in various European populations, Mueller et.al [10] reported that only two out of the four studied regions were well represented in the Hapmap CEPH population [7]. While such studies on European populations are plenty, only a few have focused on Asian populations and their concordance with the Han Chinese or Japanese Hapmap populations. A recent study looked at a 21 Mb region on chromosome 1q21-q25 in 80 Chinese Hans from Shanghai as part of the International Type 2 Diabetes 1q Consortium [14] where 3042 SNPs were identified to match with Hapmap data from the CHB population. Another study focused on the linkage disequilibrium of a region on chromosome 7p15, in Korean, Japanese, and Han Chinese samples also reports similar results[12]. These results are not surprising given that the study and reference populations were of the same ethnic origin from the same region. What is currently lacking is a similar validation on an ethnic Chinese population which is far removed from China. The Singapore Genome Variation Project recently published, compares three Singaporean populations (Chinese, Malay and Indian) against the Hapmap populations. Interestingly they showed that most Singapore Chinese were similar to southern Han Chinese [15]. There was also evidence of population sub-structure when the Hapmap Han Chinese samples were compared with samples from the northern Han Chinese population, although the data was not conclusive due to the small sample size.

In our current study we investigate the applicability of the data obtained from the Hapmap CHB population to a Singapore Chinese population using genotyping data from the Affymetrix Gene Chip Human Mapping 500 K Array Set and the Illumina Human Hapmap 550 k chip. This would also serve to validate the use of these genome wide chips in disease based genetic association studies for a Hapmap based population from a different geographical location. To supplement the whole-genome comparison, a more focused gene based analysis of genes in the highly replicated 5q31-q33 chromosomal region for asthma was performed to compare the coverage of Hapmap SNP data in the context of a case control association study.

## Results

### Correlation of SNP frequencies for Illumina 550 k Genotyping chip

**Figure 1:**
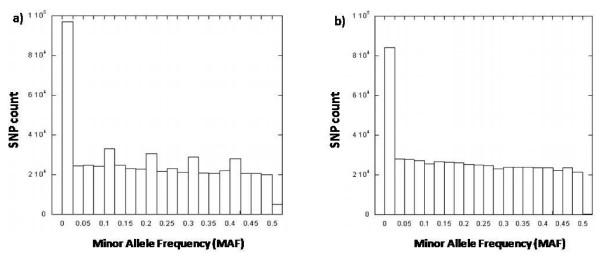
**Distribution of MAF for common SNPs in a) Hapmap CHB and b) Singapore Chinese**.

**Figure 2:**
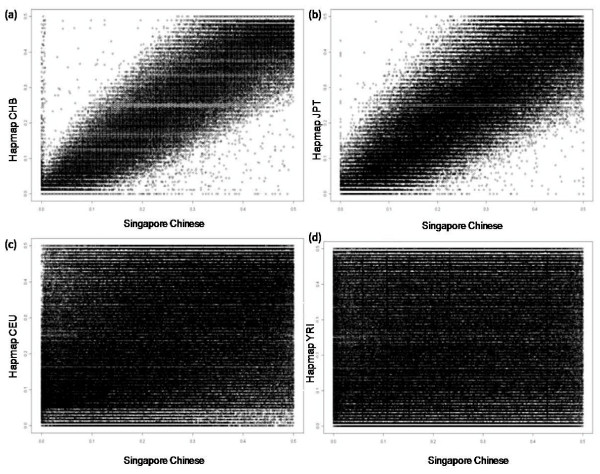
**Bi-plot of MAF in Singapore Chinese against the Hapmap populations using the Illumina 550 k chip**.

The distribution of average minor allele frequencies (MAF) for all 561466 SNPs appears to be biased towards common SNPs with more than 70% of the SNPs having a MAF of more than 0.1. While we were able to probe for all the SNPs on the Illumina 550 k chip, comprehensive genotyping data was not available for all the four Hapmap populations. As such, only the SNPs common to each of the Hapmap populations and our study population were selected and used for comparison. The tally of common SNPs and the concordance between the allele frequencies of these SNPs in the Hapmap populations with those from our study population are listed in Table 1. The distribution of MAF for the common SNPs in the Hapmap CHB population (Figure 1a) appears to be more evenly distributed than what was observed in our study population (Figure 1b). Comparing the MAF, a Pearson's correlation of 0.954 is obtained which reveals that our study population of Singapore Chinese is highly similar to the Hapmap CHB population (Figure 2a). This high concordance is confirmed by the relatively small (0.09%) proportion of the 550763 common SNPs showing a difference of more than 0.2 in MAF (Table 2). Comparing the MAF of Singapore Chinese with the other Hapmap populations, the Hapmap Japanese population (Figure 2b) remained fairly concordant with r of 0.91 with 1727 out of 356129 (0.49%) common SNPs having a difference in MAF of over 0.2. The allele frequencies from the Hapmap CEPH and YRI populations were significantly different from our study population with Pearson's correlation of 0.46 and 0.17 respectively (Figure 2c and 2d respectively).

**Table 1:** Concordance Correlation Coefficient for Hapmap populations against Singaporean Chinese population using Illumina 550 k chip

Hapmap Population	Common SNPs	Correlation
**CHB**	557063	0.95
**JPT**	356129	0.90
**CEPH**	557455	0.53
**YRI**	557063	0.21

**Table 2:** Difference in average MAF for SNPs for Singaporean Chinese population to Hapmap Han Chinese population

Difference in MAF	Number of SNPs	MAD
**0.4 - 0.5**	47 (0.01%)	0.4431
**0.3 - 0.399**	92 (0.02%)	0.3439
**0.2 - 0.299**	363 (1.4%)	0.23
**0.1 - 0.199**	25916 (4.65%)	0.1224
**0.05 - 0.099**	118249 (21.23%)	0.0688
**0 - 0.05**	412396 (74%)	0.0187

**Total**	550760	

### Correlation of SNP frequencies for Affymetrix 500 k Genotyping chip

**Figure 3:**
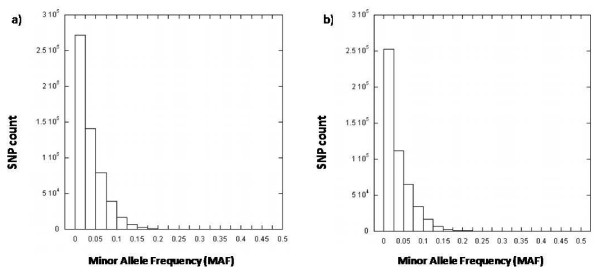
**Differences in MAF between Hapmap CHB and Singapore Chinese using a) Illumina and b) Affymetrix chips**.

**Figure 4:**
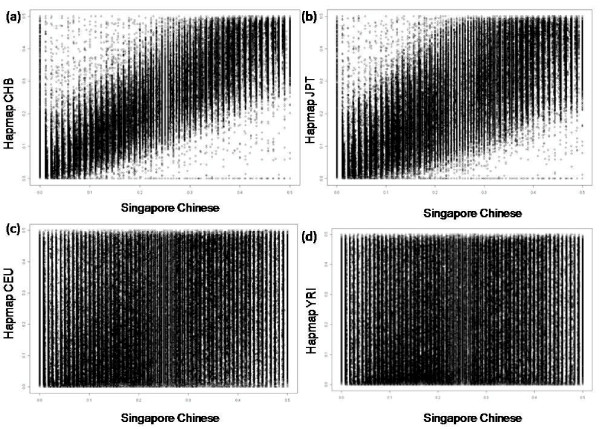
**Bi-plot of MAF in Singapore Chinese against the Hapmap populations using the Affymetrix 500 k chip**.

Minor allele frequencies for the 500 568 SNPs on the Affymetrix 500 k chip were generated and their distribution plotted. SNPs common to each of the four Hapmap populations were compared with our genotyping data and the results match closely to those obtained from the Illumina platform (Table 3). The MAF of common SNPs with the Hapmap CHB population also appear to be similarly distributed (Figure 3). While the Affymetrix 500 k Gene Chip did not perform as well as the Illumina 550 k chip, with 1286 (0.26%) of SNPs having allele frequencies differing by more than 0.2 in comparison with the Hapmap CHB population, a degree of high concordance (r = 0.947) was still evident (Table 4, Figure 4).

**Table 3:** Concordance Correlation Coefficient for Hapmap populations against Singaporean Chinese population using Affymetrix 500 k chip

Hapmap Population	Common SNPs	Correlation
**CHB**	492496	0.94
**JPT**	492496	0.89
**CEPH**	492471	0.53
**YRI**	489438	0.23

**Table 4:** Difference in average MAF for SNPs for Singaporean Chinese population to Hapmap Han Chinese population

Difference in MAF	Number of SNPs	MAD
**0.4 - 0.5**	212 (0.043%)	0.446048
**0.3 - 0.399**	376 (0.076%)	0.342023
**0.2 - 0.299**	1286 (0.2612%)	0.234257
**0.1 - 0.199**	27712 (5.627%)	0.125867
**0.05 - 0.099**	98826 (20.07%)	0.069757
**0 - 0.05**	364082 (73.93%)	0.034877

**Total**	492496	

### Chromosome based Analysis

To detect any patterns in chromosomal aggregation of similarities between Singapore Chinese and Hapmap CHB, the MAF comparison was performed at a chromosomal level. Pearson's correlation was found to be consistently high along each of the chromosomes with the lowest value being 0.948 (Additional file 1: Figure S2). This high concordance was not related to the number of SNPs from the Illumina 550 k chip on each chromosome or the length of the chromosome (Additional file 1: Figures S1, S2, and S3). Of the 561466 SNPs on the chip, 502 were found to be discordant compared to the Hapmap CHB data, with MAFs differing by up to 0.2. To identify if these SNPs were in any potential chromosomal hotspots, they were mapped to regions within each chromosome based on their physical positions. No particular chromosomal hotspot was found (Data not shown).

### Principal Component Analysis (PCA)

**Figure 5:**
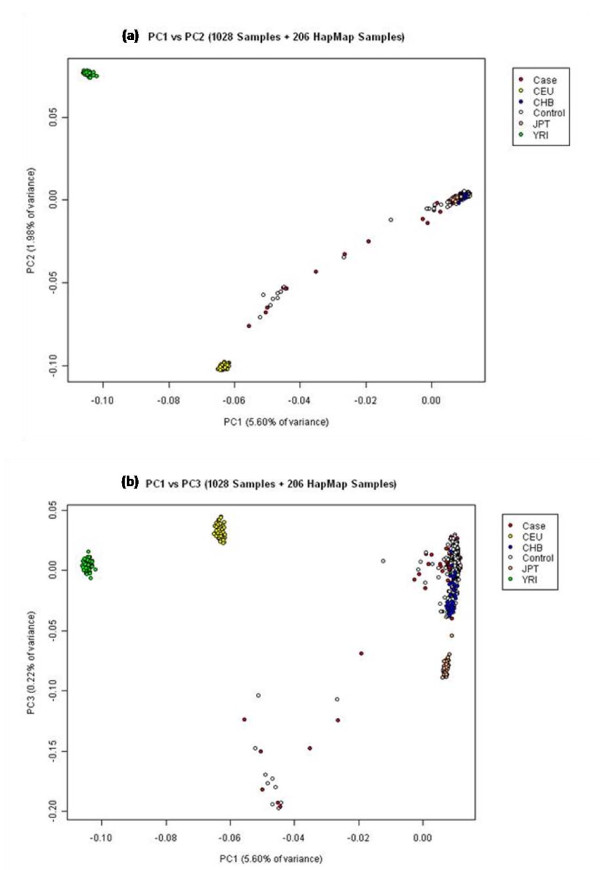
**Principal component plots for PC1 against PC2 and PC3 for 1028 Singapore Chinese and 206 Hapmap samples**.

PCA was used to evaluate the population structure of the Singapore Chinese samples in comparison to the Hapmap populations. Plots of the first five principal components were generated using data from our 1028 ethnic Chinese samples genotyped on the Illumina beadchip and from 206 Hapmap samples. The Hapmap samples used in the analysis consisted of 60 CEPH samples (Utah residents with ancestry from northern and western Europe), 57 YRI samples (Yoruba in Ibadan, Nigeria), 44 JPT samples (Japanese in Tokyo, Japan) and 45 CHB samples (Han Chinese in Beijing, China). Scatter plots of the first two components (PC1 vs PC2) show that these components clearly differentiated the Asian populations (CHB and JPT) and Singapore Chinese from the Caucasian (CEU) and Yoruba (YRI) samples (Figure 5a).

**Figure 6:**
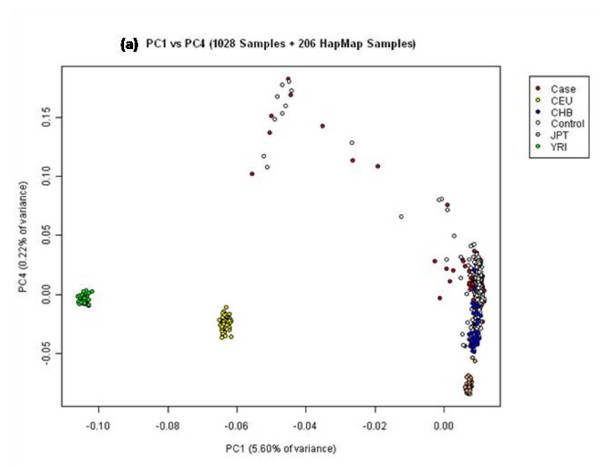
**Principal component plots for PC1 against PC4 and PC5 for 1028 Singapore Chinese and 206 Hapmap samples**.

The majority of individuals from our cohort who were classified according to self-reported ethnic identities clustered well with the Hapmap Han Chinese samples. Scatter plots of the third, fourth and fifth dimensions (PC3, PC4, PC5) progressively revealed the differences between the Chinese and the Japanese samples (Figure 5b and Figure 6a and 6b). Although the Chinese and Japanese samples are considered to be comparable in terms of Linkage Disequilibrium (LD) we observed in the plot of PC1 vs PC5 that these samples also show differences in population structure.

**Figure 7:**
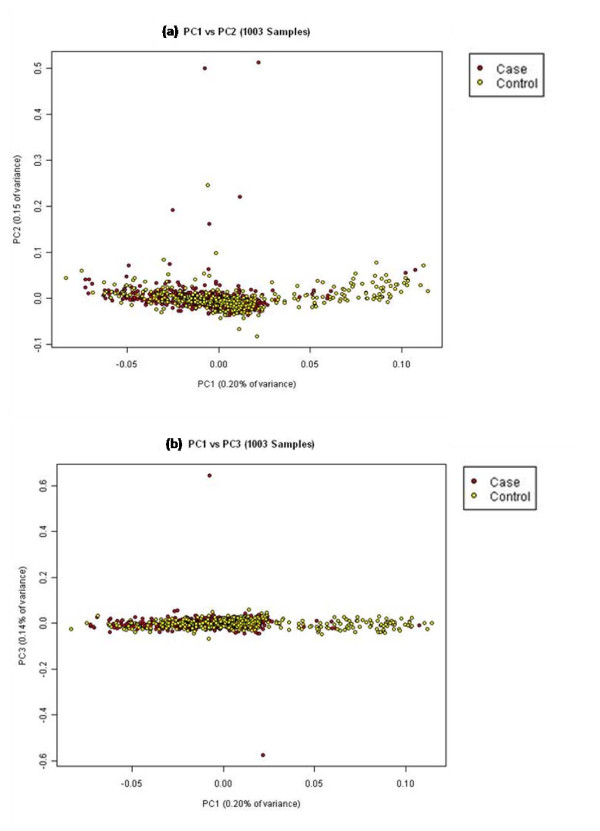
**Principal component plots for PC1 against PC2 and PC3 for 1001 Singapore Chinese samples**.

**Figure 8:**
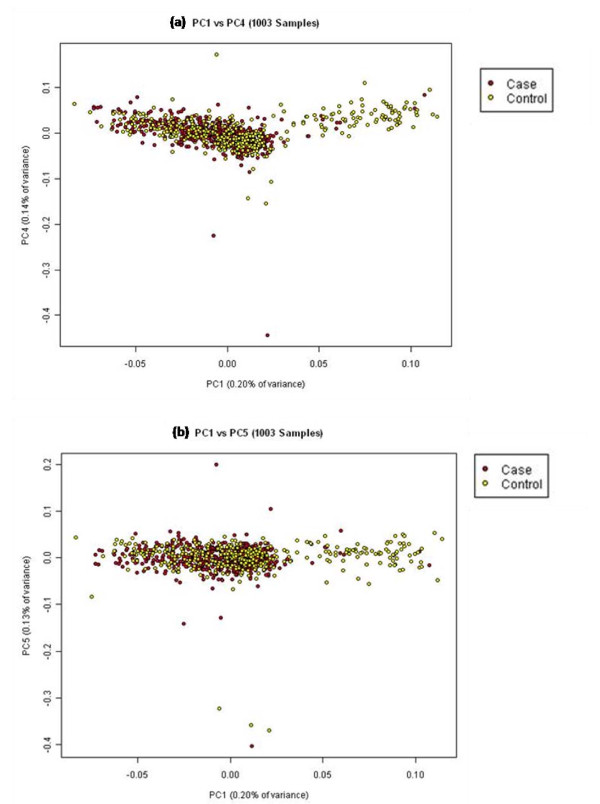
**Principal component plots for PC1 against PC4 and PC5 for 1001 Singapore Chinese samples**.

Of the 1028 ethnic Chinese samples in our cohort, 1003 samples were used for subsequent statistical analyses after removing 25 samples which were observed to be outliers. The scatter plots of the first and up to the fifth principal components (Figure 7 and Figure 8) showed that our study population was largely homogenous with no significant evidence of population stratification amongst the case and control groups. However, there still appeared to be 83 samples (8%) which drifted away from the main cluster. In tracing back the possible reasons for this drift, we discovered that while they were indeed of ethnic Chinese origin, they were born in China unlike all our other Chinese samples those who were born in Singapore. We believe this difference can be explained by the fact that Singapore Chinese are largely descendents of immigrants from southern China [15] whereas those 83 samples were likely to have originated from northern China. A plot containing the Singapore Chinese samples against the Hapmap Han Chinese only supported the above mentioned results (Additional File 1: Figure S4)

### Identification of SNPs by resequencing Asthma Candidate Genes

**Figure 9:**
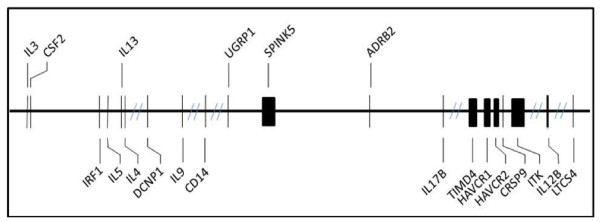
**Twenty candidate genes for asthma and atopy on chromosome 5q31-33**.

To investigate the applicability of the Hapmap CHB population to our Singapore Chinese population at the gene level, 20 genes from the 5q31-q33 region (Figure 9) which was previously found to be associated with asthma [16], as well as two genes outside of this region were re-sequenced as part of an ongoing association study. All exons, introns containing exon-intron junctions, and up to 0.5-kb promoter regions were re-sequenced, on 40 unrelated Singapore Chinese individuals. A total of 237 genetic variants were identified distributed proportionately among the 20 genes. Minor allele frequency calculations showed that the majority of the SNPs (198 or 85%) were common (MAF >0.05). In the context of a candidate gene study, the identification of common SNPs specific to the study population aids in the selection of SNPs for genotyping and subsequent association testing.

### SNP Coverage of 5q31-33 Region in Public Databases

The 237 SNPs detected in this study were evaluated against the Hapmap and dbSNP databases to compare their coverage of the 5q31-q33 region. A significantly lower number of SNPs were found in these two databases with dbSNP providing information for about 74% and Hapmap about 40% of the SNPs (Table 5). However an additional 24% SNPs had a proxy in Hapmap.

**Table 5:** SNP coverage for the 20 studied genes on 5q31-33 in various Hapmap populations

Hapmap Population	Hapmap SNPs
**ASW**	60
**CEU**	92
**CHB**	94
**JPT**	94
**YRI**	93
**CHD**	61
**MKK**	57
**TSI**	61
**MEX**	60
**GIH**	61
**LWK**	58

### Microarray Coverage

The resequencing data was used to estimate the microarray coverage of the Illumina 550 k chip for the 20 genes resequenced. Out of the 237 SNPs identified, only 182 were reported previously and 52 have not been documented previously. Thus only the previously reported SNPs were used to estimate coverage of the whole genome chip.

It was identified that only 13% of the SNPs were present on the chip, however the coverage increased to 71% if we considered SNPs that were covered by these SNPs at an r^2 ^value of 0.8. This coverage increased even further to 86% for a r^2 ^value of 0.5. (Table 6)

**Table 6:** Microarray coverage for Singapore Chinese population for the region containing the 20 genes resequenced on 5q based on Illumina 550 k chip

	Coverage (%)
**SNPs on microarray**	13
**r^2 ^> = 0.8**	71
**r^2 ^> = 0.5**	86

### Linkage Disequilibrium Analysis

**Figure 10:**
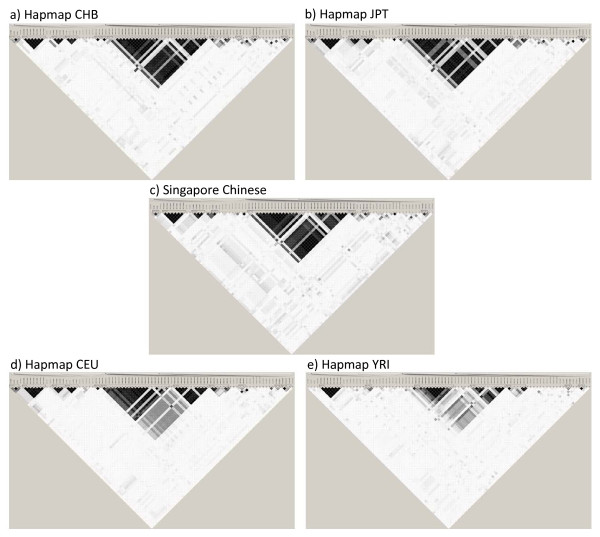
**Linkage disequilibrium pattern for 5q31-33 region in Singapore Chinese and four Hapmap populations**.

Given the small number of common SNPs within the 5q31-q33 region which were available in the SNP databases, allele frequency comparisons would not have been meaningful. However, patterns of linkage disequilibrium would be an indication of whether the available SNPs were sufficient to represent the gross variation within the chromosomal region. 15 blocks of linkage were identified from the 237 SNPs genotyped in our Singapore Chinese population. LD blocks for the common SNPs from dbSNP were then generated for the four Hapmap populations and Singapore Chinese (Figures 10a, b, c, d, and 10e). Comparing these LD patterns to what was obtained from our targeted re-sequencing; it was obvious that the SNP coverage in existing SNP databases is not comprehensive enough at this point in time for us to decipher the patterns of variation in different populations.

We also looked at all SNPs on chromosome 5 on the Illumina 550 k whole genome chip and estimated pair wise LD values for Singapore Chinese and all the 4 hapmap populations. Additional File 1: Table S1 shows further evidence that the Singapore Chinese population is most closely associated with the Hapmap Han Chinese than the other 3 hapmap populations.

### Correlation of SNP frequencies

**Figure 11:**
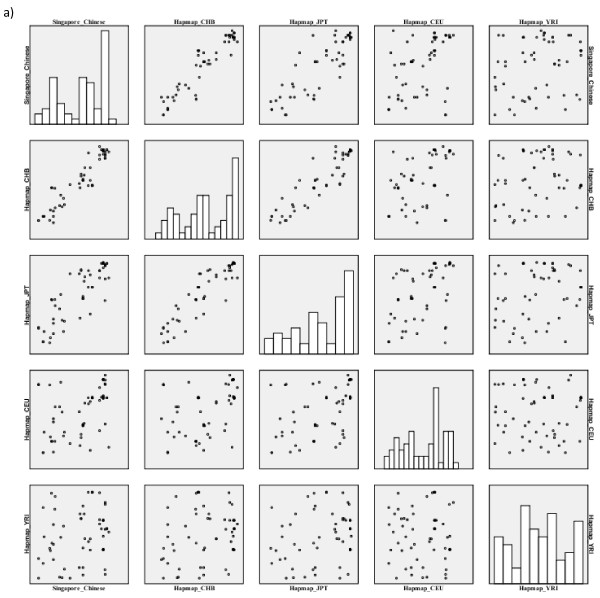
**Pair-wise correlation of Singapore Chinese against Hapmap populations from Phase I, II**.

**Figure 12:**
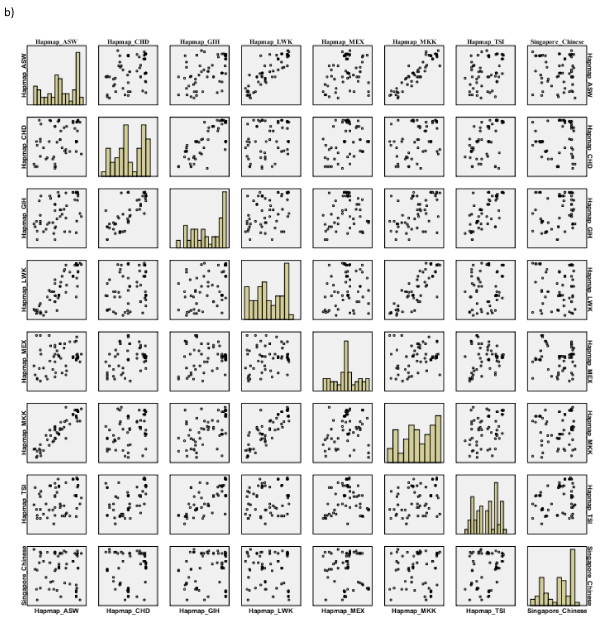
**Pair-wise correlation of Singapore Chinese against Hapmap populations from Phase III**.

We also performed a comparison of the MAF of the 11 Hapmap populations for the SNPs identified through sequencing as discussed above. There were 60 SNPs that were common across all the 11 Hapmap populations and Singapore Chinese. A scatter plot matrix was plotted to observe the correlations in the allele frequencies across these populations (Figures 11 and Figure 12). The correlation patterns were similar to those obtained from the genome-wide comparison. Singapore Chinese samples correlated well with the Hapmap CHB population and to a lesser extent with the Hapmap JPT samples. An important observation was that, on comparison with the other 7 populations added in Hapmap Phase III, no significant correlation with the Singapore Chinese samples was observed (Table 7).

**Table 7:** Correlation of SNP frequencies between Singapore Chinese and Hapmap populations

Hapmap Population	Correlation
**CHB**	0.933
**JPT**	0.860
**CEU**	0.467
**YRI**	0.225
**ASW**	0.065
**CHD**	0.122
**GIH**	0.199
**LWK**	0.079
**MEX**	0.117
**MKK**	0.068
**TSI**	0.286

## Discussion

Genes underlying common complex diseases - such as asthma and other allergic diseases, are likely to be multiple, each with a relatively small effect, but act in concert or with environmental influences to lead to clinical presentation [17]. The Hapmap project was designed to allow researchers to identify common disease-causing variants based upon the "common disease, common variant" hypothesis, which suggests that genetic influences on many common diseases are attributable to a limited number of allelic variants (one or a few at each major disease locus) that are present in more than 1-5% of the population [18-20]. Linkage Disequilibrium (LD) data is also available in Hapmap to facilitate the design of genome wide chips for association studies. This study attempts to explore the genetic architecture of the Singapore Chinese population. By considering our population in the context of the Hapmap populations, this study reveals significant insights that are relevant in conducting genetic studies in a population of Chinese ancestry.

### Hapmap data and the world populations

Hu et.al., [8] had described that Shanghai Chinese are very similar to Hapmap Han Chinese based on 4,500 SNPs in a 21 Mb region on chromosome 1q21-q25 in 80 unrelated Shanghai Chinese and 45 Hapmap Beijing Han Chinese. They had a correlation coefficient of R = 0.94, p < 0.001 for 3042 SNPs (some SNPs were filtered out based on their data quality control criteria). They also reported a similar correlation coefficient of R = 0.88, p < 0.001 for comparison of Shanghai Chinese to Hapmap Japanese. Takeuchi et.al [21] performed a similar comparison of Japanese individuals against Hapmap Japanese by combining resequencing and high-density genotyping approaches. They stated that the Hapmap coverage is not thorough for SNPs in the Japanese population, and this needs to be considered when association results are interpreted. Researchers elsewhere have also performed comparative studies between CEU SNP data and several other populations, including Spanish, Finnish, and Estonia [10,12,13,22]. They all came to the same conclusion that the CEU SNP dataset was a robust dataset for comparative and association studies in these populations. These various observations by different groups studying the effectiveness of Hapmap dataset for different populations were not really consistent. Even though Hapmap serves as a good reference population for some populations, its applicability to other populations not evaluated in the Hapmap project needs to be assessed closely.

### Hapmap and Singapore Chinese

The genotype data for Singapore Chinese from both Illumina and Affymetrix have given us a high correlation coefficient of 95% in comparison to the Hapmap Han Chinese. On the contrary, comparison with the Caucasian and African populations showed very low correlation. However in a comparison of close to 1 million SNPs, 5% deviance is still somewhat significant. In an attempt to localize this deviation, a chromosome based correlation analysis was performed. A consistently high correlation (more than 95%) was observed across all the chromosomes with deviating SNPs not associated with minor allele frequencies or any specific chromosomal location. This indicates that the 5% deviation observed between Hapmap CHB and our local population was likely to be random and not due to any major differences in the two populations.

The HumanHap550 BeadChip from Illumina displays a genomic coverage 87% for the Asian population (CHB+JPT) and 90% and 57% for the CEU and YRI populations respectively (Illumina Inc) as measured by Phase I+II Hapmap genotype data. The mean MAFs determined using the HumanHap550 BeadChip was 0.23, 0.21 and 0.22 for the CEU, CHB+JPT and YRI populations, respectively [7]. It should be noted that though the mean MAF is similar for all 3 populations, the distribution of SNPs in terms of MAF is quite different. The mean MAF determined for our Singapore Chinese population is 0.215 which is similar to the estimates for Asian population as reported by Illumina. The high genomic coverage for the Asian population set, as well as the comparable mean MAF suggest that the BeadChip designed based on linkage disequilibrium data from Hapmap can be extended for genome-wide analysis of other similar population cohorts not previously genotyped, such as the Singapore Chinese in our case. A very recent study by Chen et.al [23] describes the genetic architecture of Han Chinese from all over the world and found a ''north-south'' population structure which was also clearly visible in our population. The study had also included 570 Han Chinese samples from Singapore and found they were more similar the southern Han Chinese population. These differences need to be addressed while performing association studies including samples from both northern and southern Han Chinese samples in the same study.

Using the resequencing data of the 5q31-33 region, we compared and estimated the coverage of the genes in this region with that in the Hapmap project. Of the 237 SNPs we identified through resequencing; 73 (31%) were identified in Hapmap. This meant that more than two-thirds of the variation in Singapore Chinese was not reported in the Hapmap CHB population. While a further 24% of our 'novel' SNPs had proxies in Hapmap, the fact remains that even the Hapmap CHB population, likely to be genealogically closest to Singapore Chinese, was unable to provide information for at least 50% of the genetic variation in our local population. In the study of complex diseases, such as asthma, it is of the utmost importance to capture as much of the genetic variation in the study population as possible so that they can be screened for potential associations. In such situations, Hapmap by itself may be insufficient and targeted resequencing may be essential to capture all the variation in a specific population. A study by Tantoso et al [24] has also demonstrated that the Hapmap SNPs are not robust enough to capture the untyped variants for most of genes. They estimated a marginal coverage of about 55% for European and Asian samples and the coverage is as low as 30% for the Hapmap YRI panel. A recent study also evaluated the coverage of different SNP chips used for genome-wide association studies [11]. Such information would be useful in selecting the chip which would provide a better coverage for the population under investigation.

## Conclusions

In this study we evaluated the correlation between MAF of Hapmap SNPs and that obtained from a Singapore Chinese population. We found that minor allele frequencies of 976219 unique Hapmap SNPs for the Han Chinese population correlated with those from a Singapore Chinese population with a concordance correlation coefficient of 0.95. This clearly demonstrates the effectiveness of using Hapmap Han Chinese population as a reference population for future whole genome based association studies in Singapore Chinese. It also emphasizes the fact that the SNPs selected in the Genome wide chips are performing as expected as the MAF are quite similar to the actual MAF in the Hapmap project. Although the principal component analysis reveals no significant population stratification, the migration pattern of the samples needs to be addressed while designing and interpreting genome-wide association studies. While we showed that the SNPs deposited in Hapmap are sufficient to represent the gross genetic variation based on the similar LD patterns observed between both Hapmap Han Chinese and Singapore Chinese populations, targeted resequencing, as used in a candidate gene based approach, may still be necessary to capture all the variation in specific target genes. This SNP information can also help to develop SNP chips which are more targeted towards a specific population which clear population signatures.

## Methods

### Samples

The DNA samples used in this study were collected from ethnic Chinese participants following standard protocols of informed consent, as part of an on-going whole-genome asthma and allergic disease case-control association study (unpublished data) in Singapore. Experimental research that is reported in the manuscript has been performed with the approval of NUS Institutional Review Board (IRB) Reference - NUS07-023 and is also in compliance with the Helsinki declaration. Genomic DNA was extracted from buccal cells obtained from a mouthwash of 0.9% saline solution following a standardized protocol[25] In short, the buccal cells were pelleted and lysed; DNA was extracted using the phenol-chloroform phase-separation technique [22] purified by two washes in ethanol, with the DNA pellet resuspended in reduced Tris-EDTA buffer. Samples were quantified in triplicate on the Nanodrop (ND-1000). Samples which fell within a 1% error margin in the replicate measurements were subsequently diluted to 50 ng/μl, according to the requirements in the assay manual.

### Genotyping

A total of 114 and 1028 samples were genotyped on the Affymetrix 500 k and Illumina Hapmap 550 k chips (Illumina Infinium HumanHap550 Duo or Illumina Infinium HumanHap610 Quad) which were processed according to the protocol outlined in the Gene Chip Mapping 500 k Assay Manual and Infinium II Assay Workflow respectively. Genotypes were obtained using the BRLMM algorithm as implemented in the Genotyping Console v2.1 for the Affymetrix platform, and from the BeadStation software for the Illumina platform. Cryptic relatedness was tested to remove any relatives within the samples and gender test was also performed to ensure all predicted sexes matched the actual gender.

### Statistical Analysis

Concordance correlation coefficient was calculated to determine "correlation" as a measure of accuracy between actual and estimated allele frequencies. R software package and PASW statistics 17(SPSS Inc) were used to calculate the correlation statistics. Unless otherwise stated, all measures of correlation were deemed statistically significant at p < 0.05. Mean absolute deviation (MAD) was used as a more robust estimator of dispersion of errors than standard deviation or variance. Principal Component Analysis (PCA) statistics were calculated using the EIGENSTRAT software package[26]. LD blocks were developed using Haploview version 4.0 http://www.broad.mit.edu/mpg/haploview. The r^2 ^values were used to determine pair-wise linkage using the default Gabriel et al. algorithm. A "proxy SNP" is defined as a SNPs which is covered by another SNP at an r^2 ^value of 0.8. Microarray coverage was calculated as described by Magi et.al [27]

## Authors' contributions

AKA performed the statistical analysis and drafted the manuscript. RA genotyped samples using the Affymetrix 500 k array and edited the manuscript. PPN re-sequenced the genes for candidate gene based study for asthma. WDY edited the manuscript. FTC conceived, designed and planned the study and edited the manuscript. All authors have read and approved the final manuscript.

## Supplementary Material

Additional file 1 Supplementary Figure S1: Frequency of SNPs in each chromosome for Illumina Hapmap 550 k chip Supplementary Figure S2: Correlation Plots for each chromosome 1-22 and X. Supplementary Figure S3: Intra-chromosomal Analysis (SNPs with a difference in MAF of greater than 0.1) Supplementary Figure S4: Principal component plots for PC1 against PC2 for 1001 Singapore Chinese samples and 45 Hapmap Han Chinese samples. Supplementary table: Supplementary Table S1: Concordance correlation coefficients for r^2 ^values estimated between Singapore Chinese and other hapmap populations for SNPs on Chromsome 5. Click here for file
