# Supplementary material for: Evaluating the transferability of Hapmap SNPs to a Singapore Chinese population
Source: BMC Genet. 2010 May 7;11:36. doi: 10.1186/1471-2156-11-36 (PMC2877651; doi:10.1186/1471-2156-11-36)
Supplement: Additional file 1 — Supplementary Figure S1: Frequency of SNPs in each chromosome for Illumina Hapmap 550 k chip Supplementary Figure S2: Correlation Plots for each chromosome 1-22 and X. Supplementary Figure S3: Intra-chromosomal Analysis (SNPs with a difference in MAF of greater than 0.1) Supplementary Figure S4: Principal component plots for PC1 against PC2 for 1001 Singapore Chinese samples and 45 Hapmap Han Chinese samples. Supplementary table: Supplementary Table S1: Concordance correlation coefficients for r2 values estimated between Singapore Chinese and other hapmap populations for SNPs on Chromsome 5. [file 1471-2156-11-36-S1.DOC]

**Supplementary Figure S1: Frequency of SNPs in each chromosome for Illumina Hapmap 550k chip**


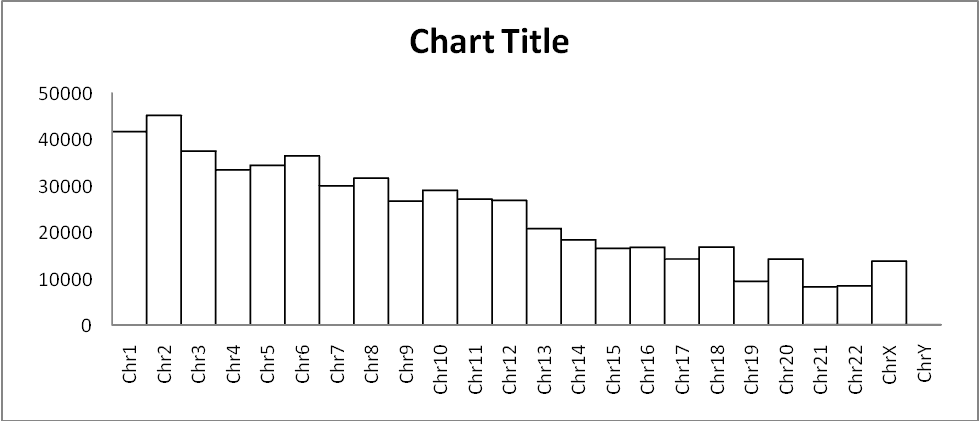


**Supplementary Figure S2: Correlation Plots for each chromosome 1-22 and X.**

**
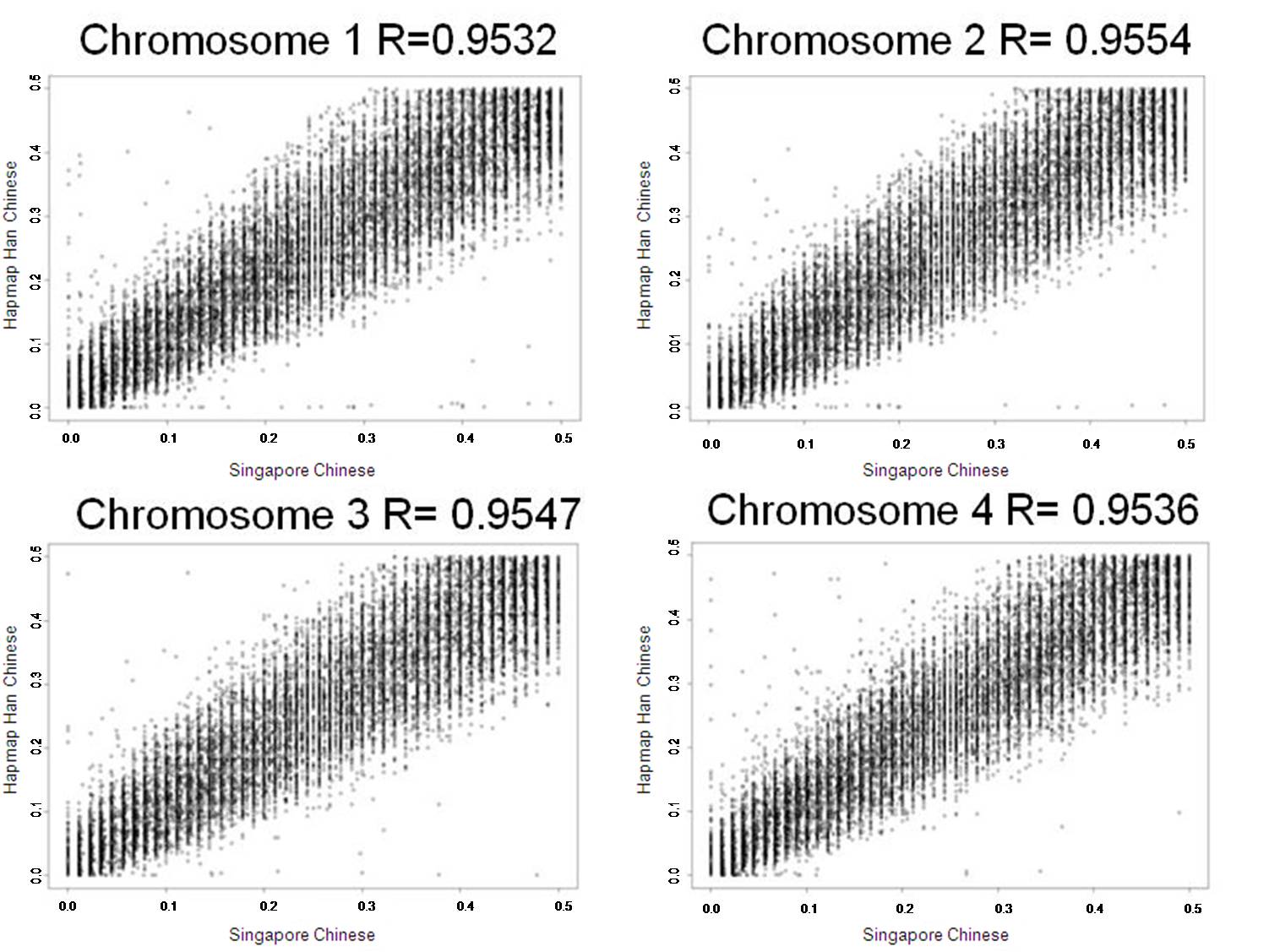
**


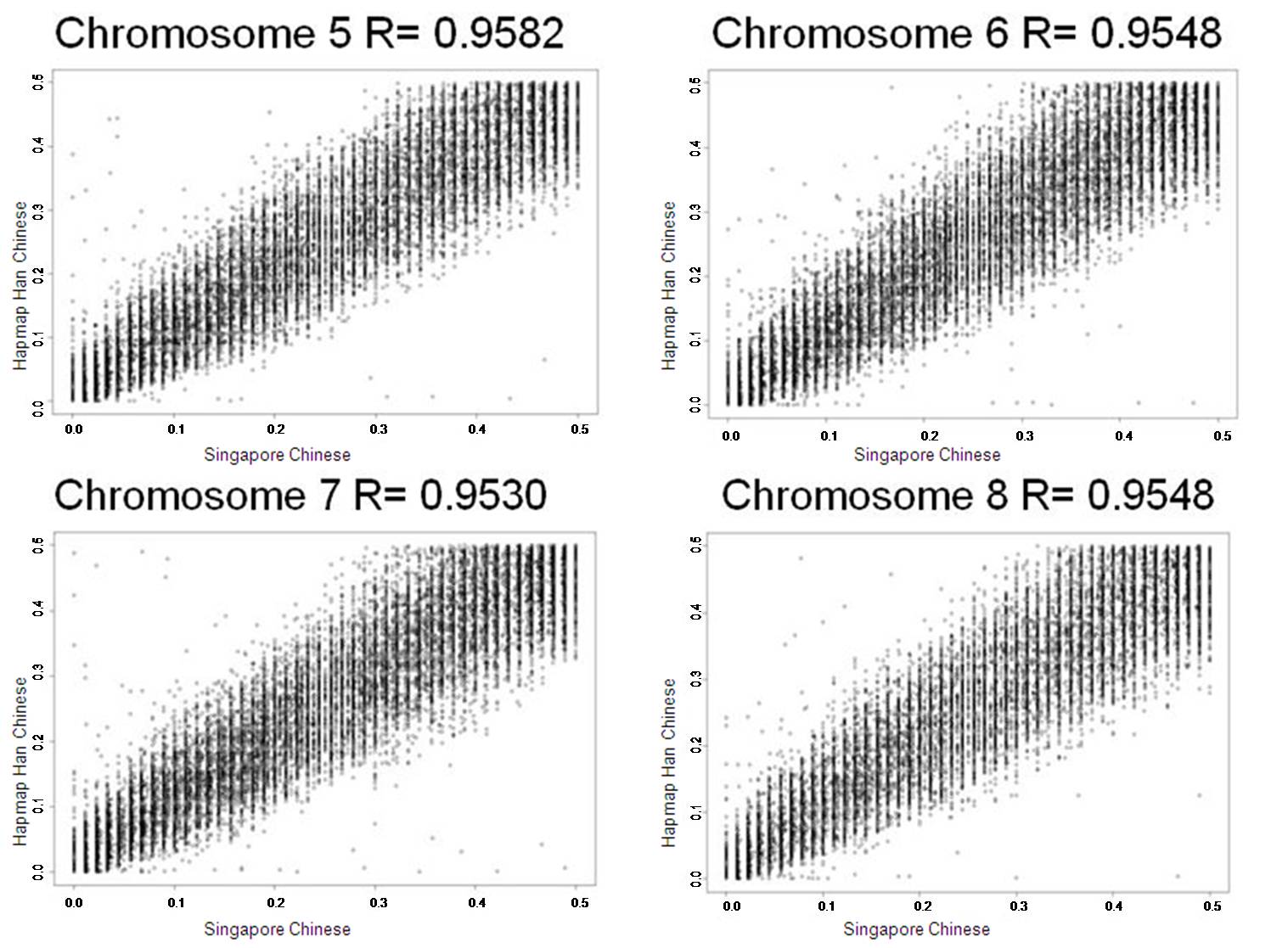


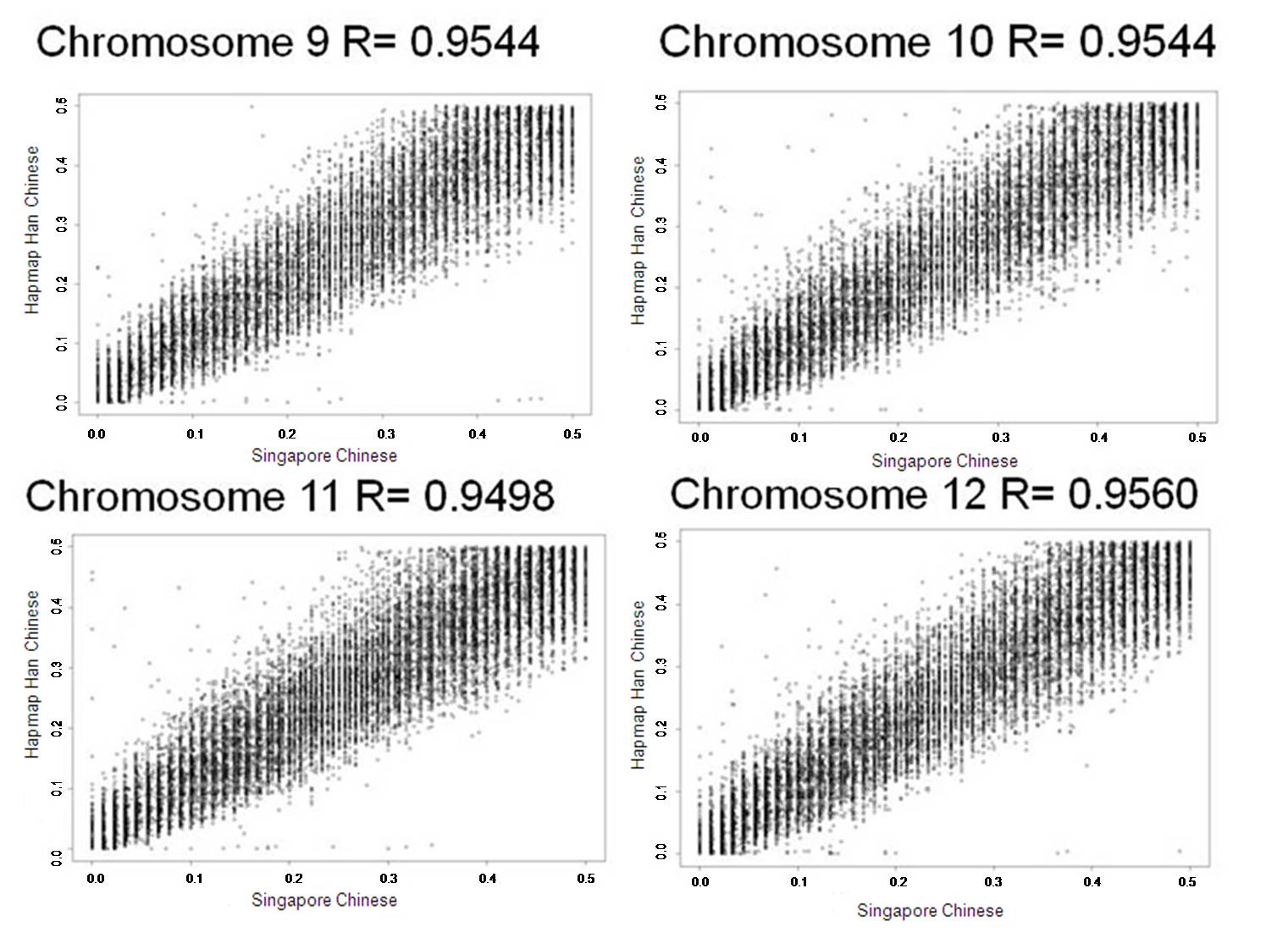


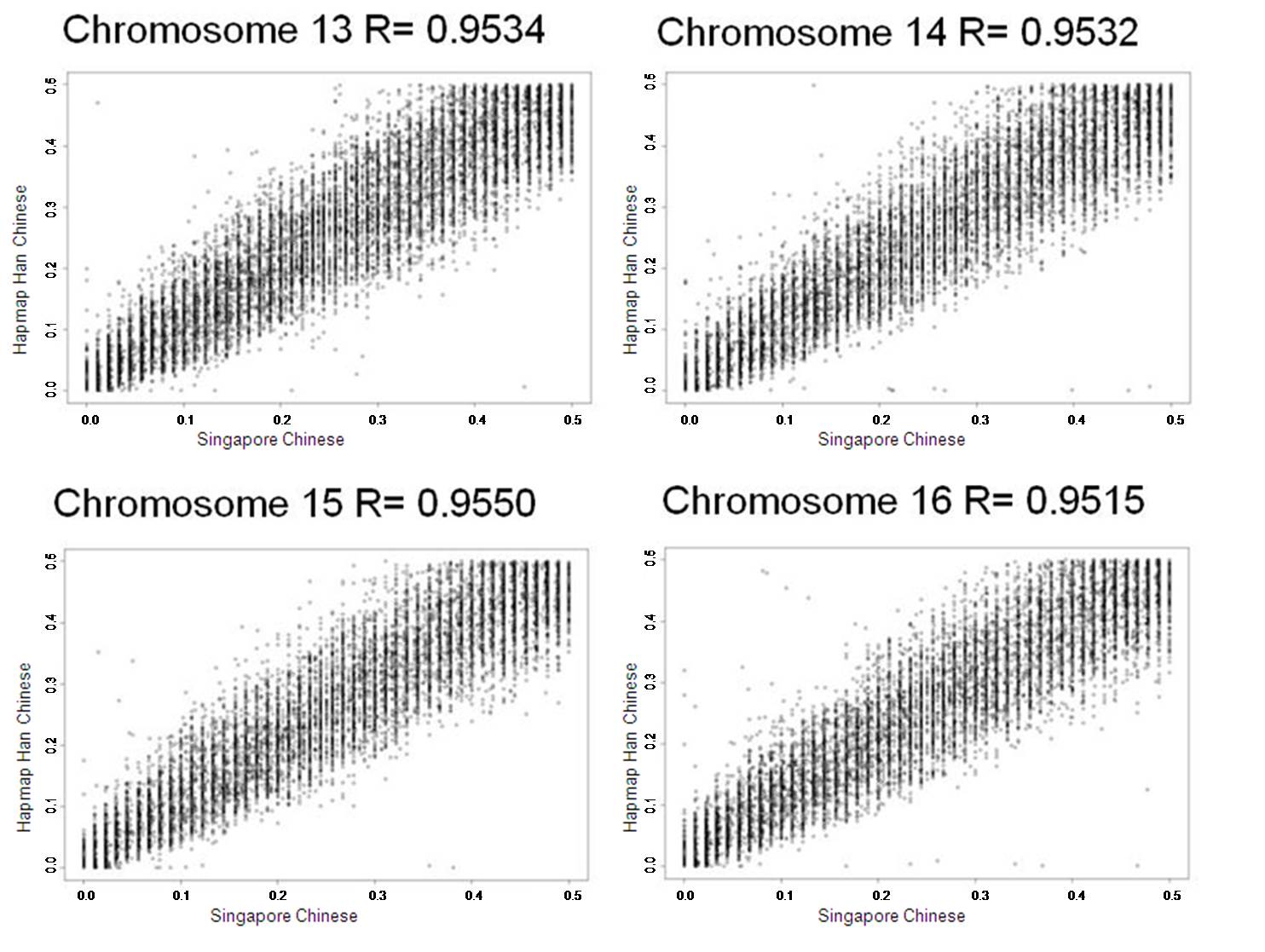


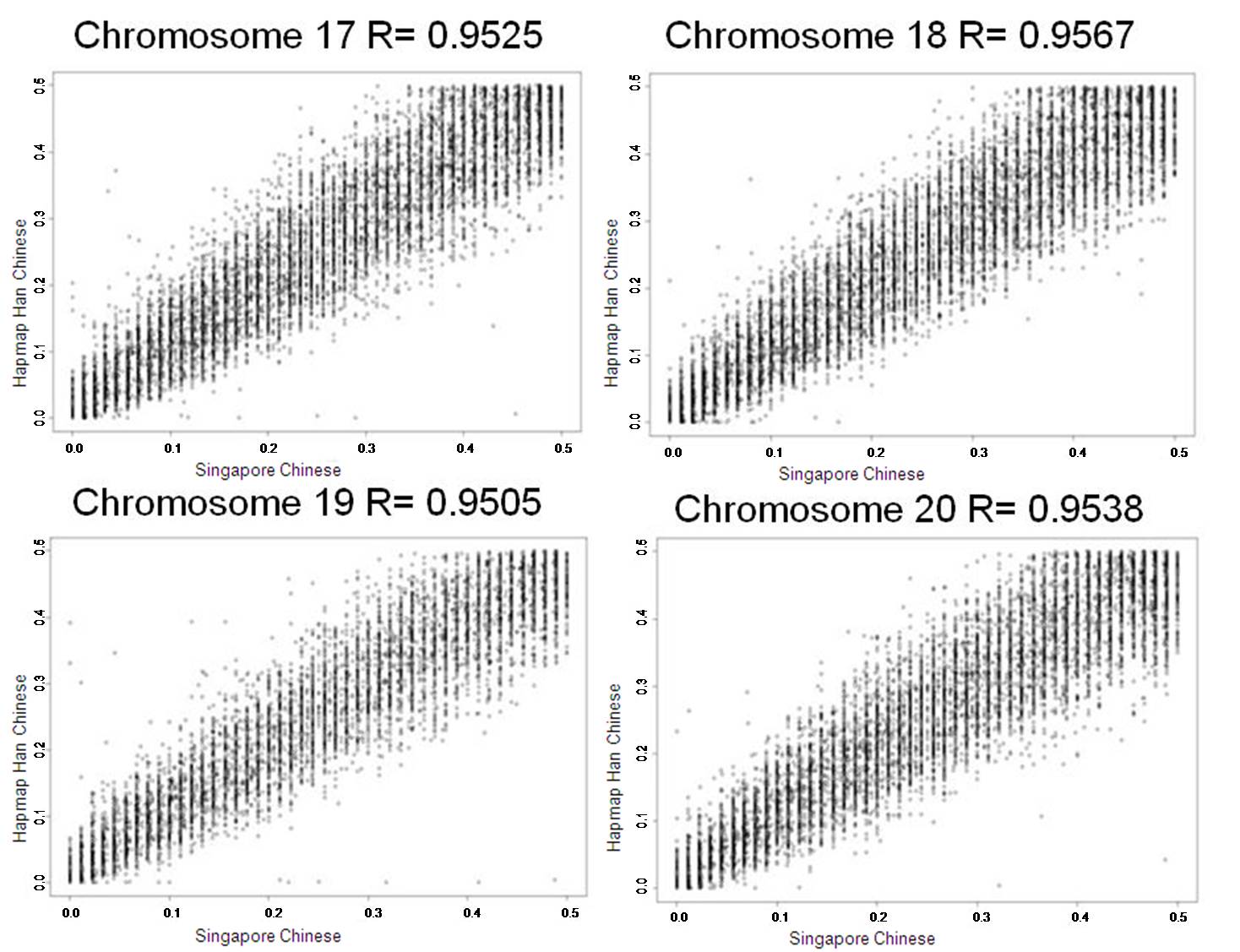


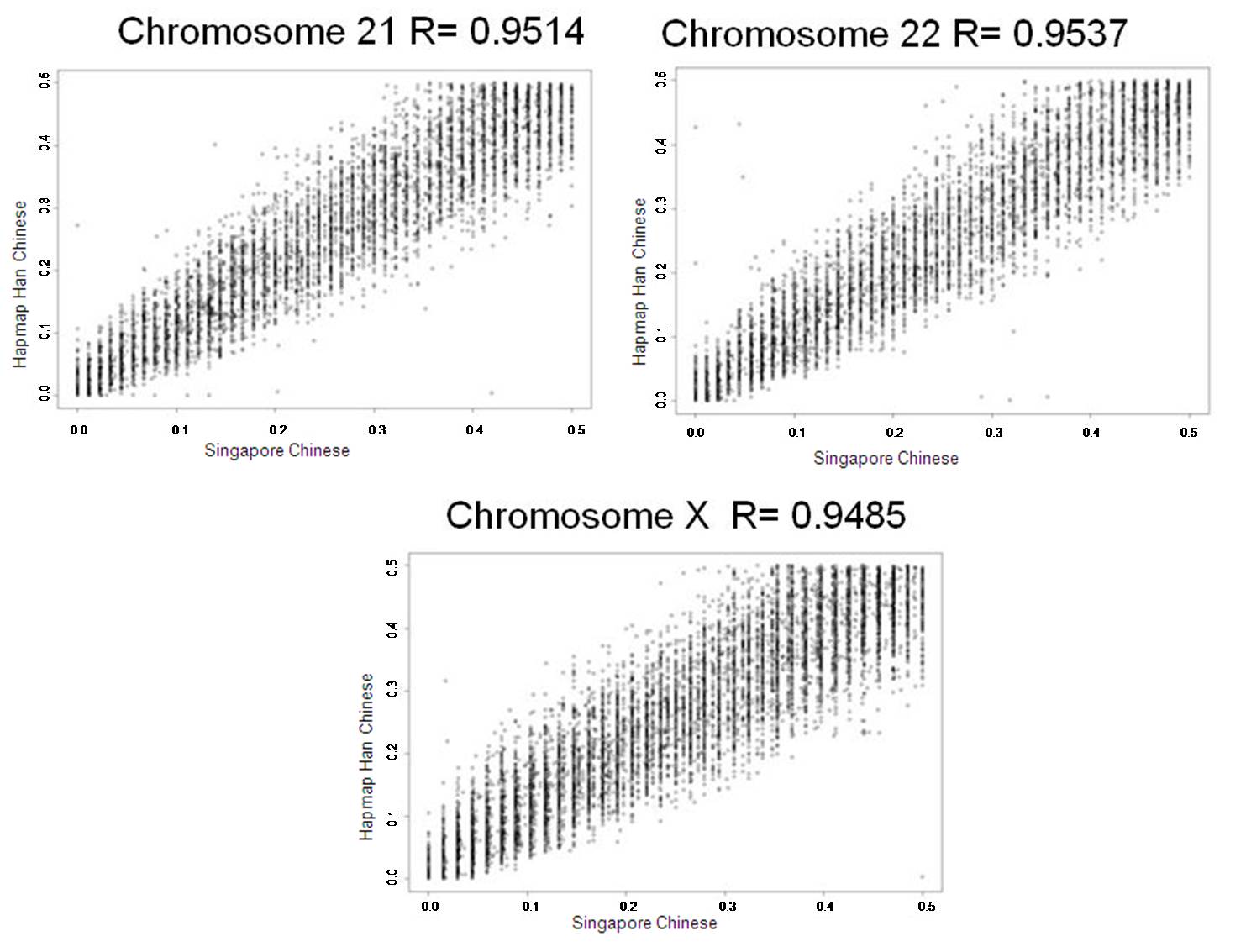


**Supplementary Figure S3: Intra-chromosomal Analysis (SNPs with a difference in MAF of greater than 0.1)**

**Chromosome 1**


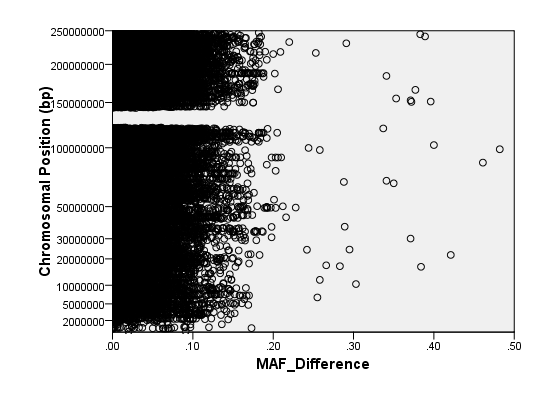


**Chromosome 2**


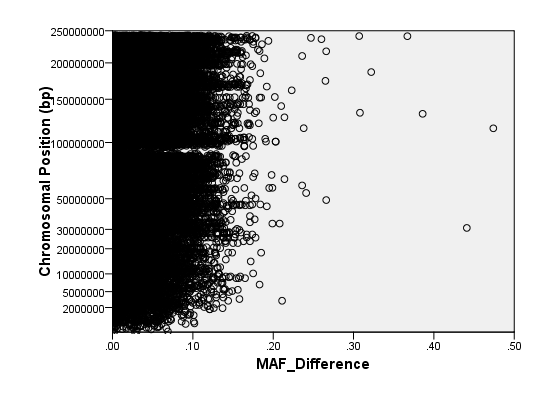


**Chromosome 3**


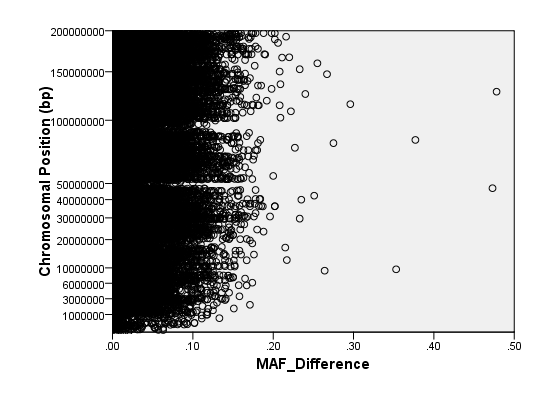


**Chromosome 4**


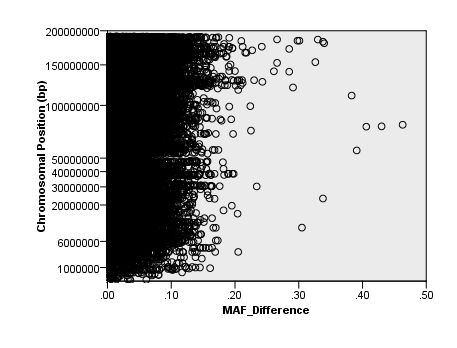


**Chromosome 5**


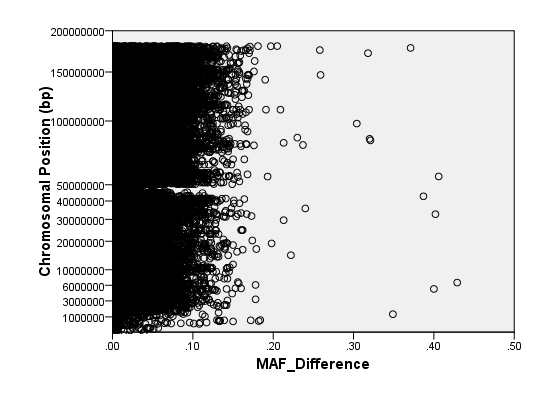


**Chromosome 6**


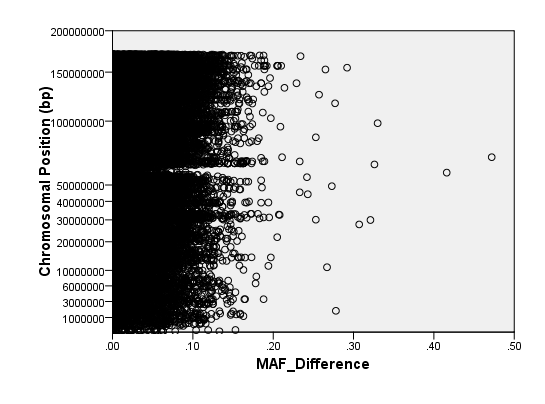


**Chromosome 7**


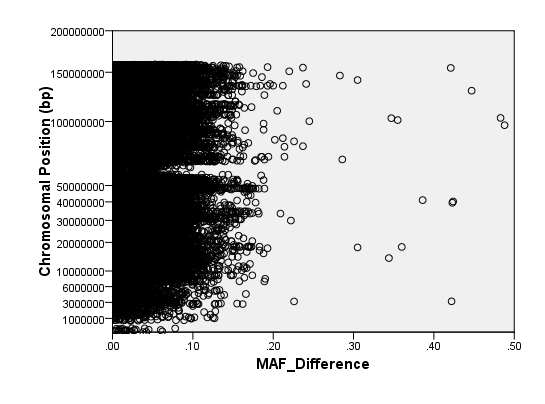


**Chromosome 8**


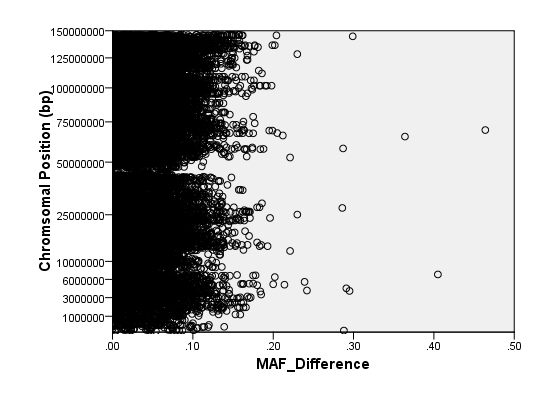


**Chromosome 9**


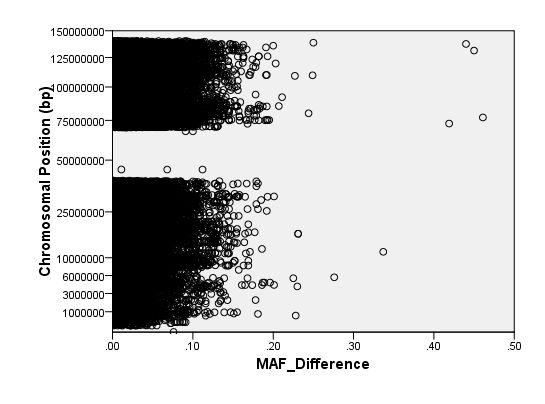


**Chromosome 10**


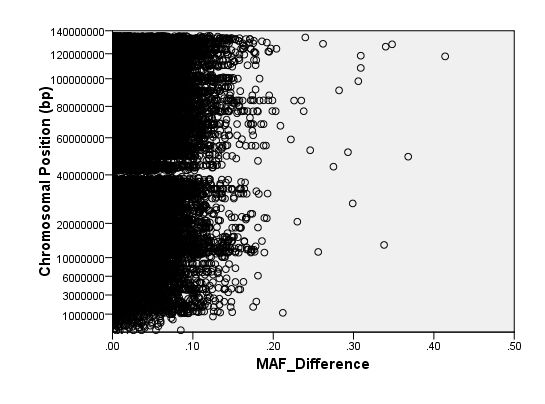


**Chromosome 11**


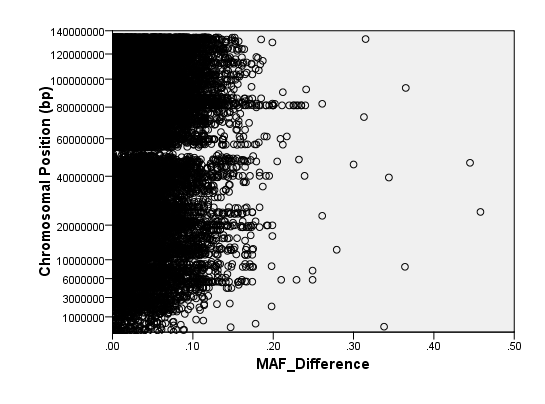


**Chromosome 12**


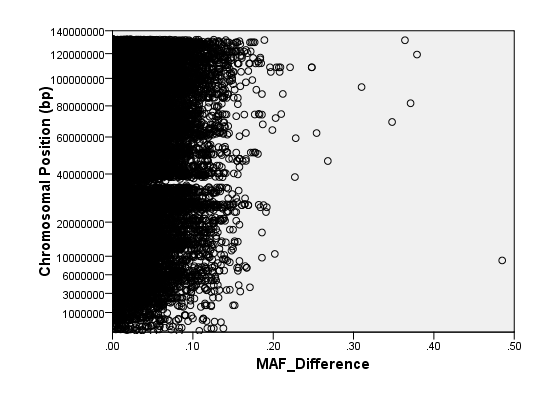


**Chromosome 13**


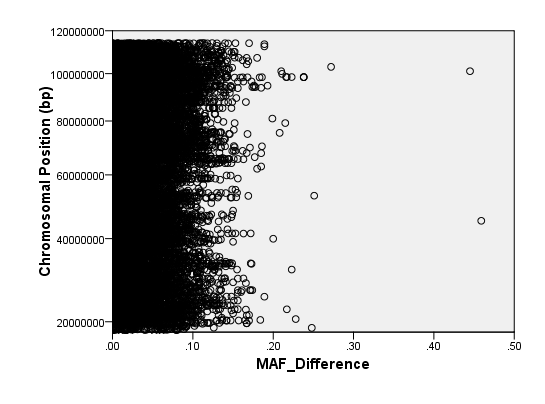


**Chromosome 14**


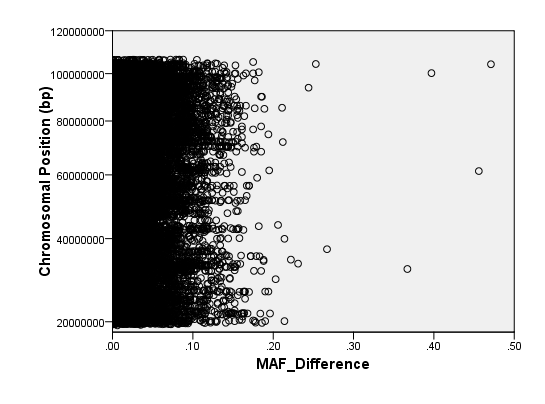


**Chromosome 15**


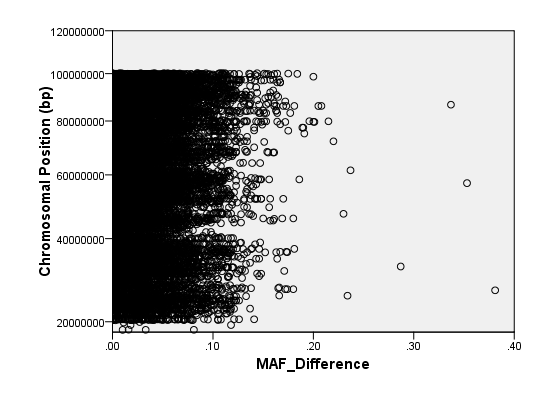


**Chromosome 16**


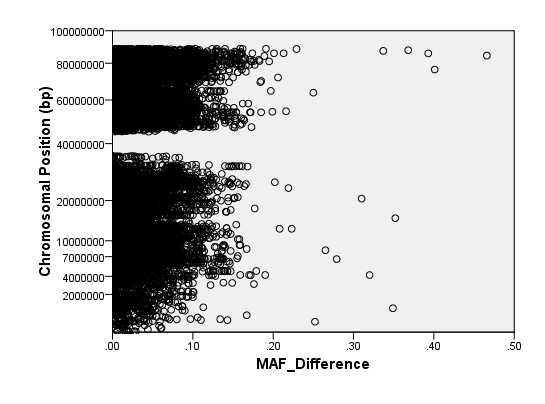


**Chromosome 17**


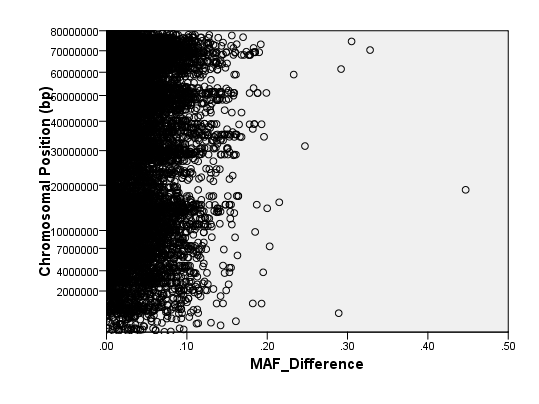


**Chromosome 18**


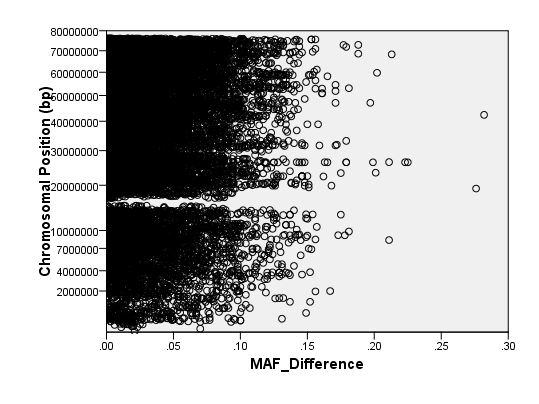


**Chromosome 19**


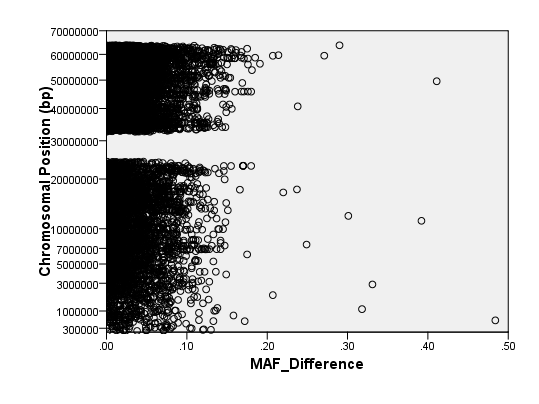


**Chromosome 20**


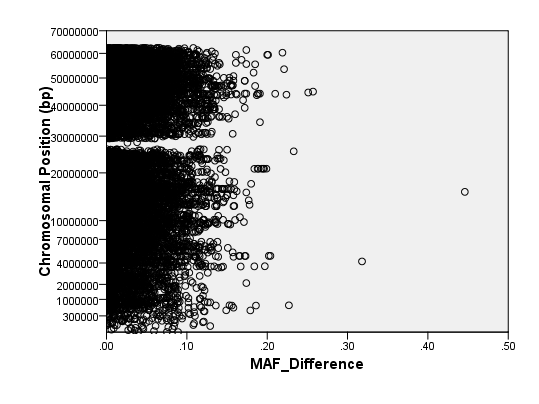


**Chromosome 21**


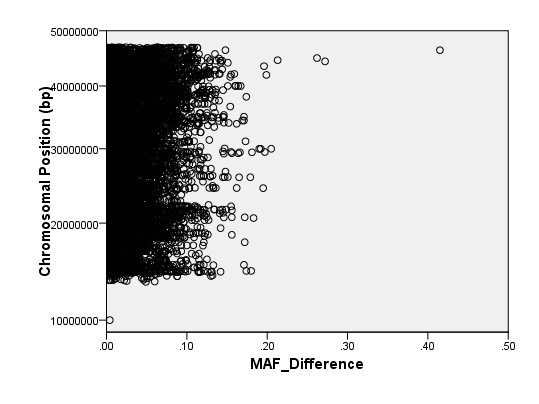


**Chromosome 22**


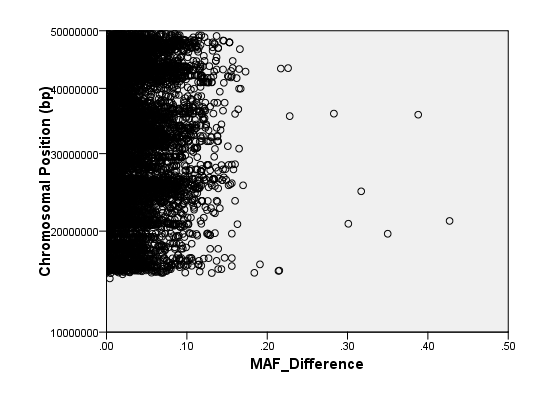


## Supplementary Figure S4: Principal component plots for PC1 against PC2 for 1001 Singapore Chinese samples and 45 Hapmap Han Chinese samples.

##
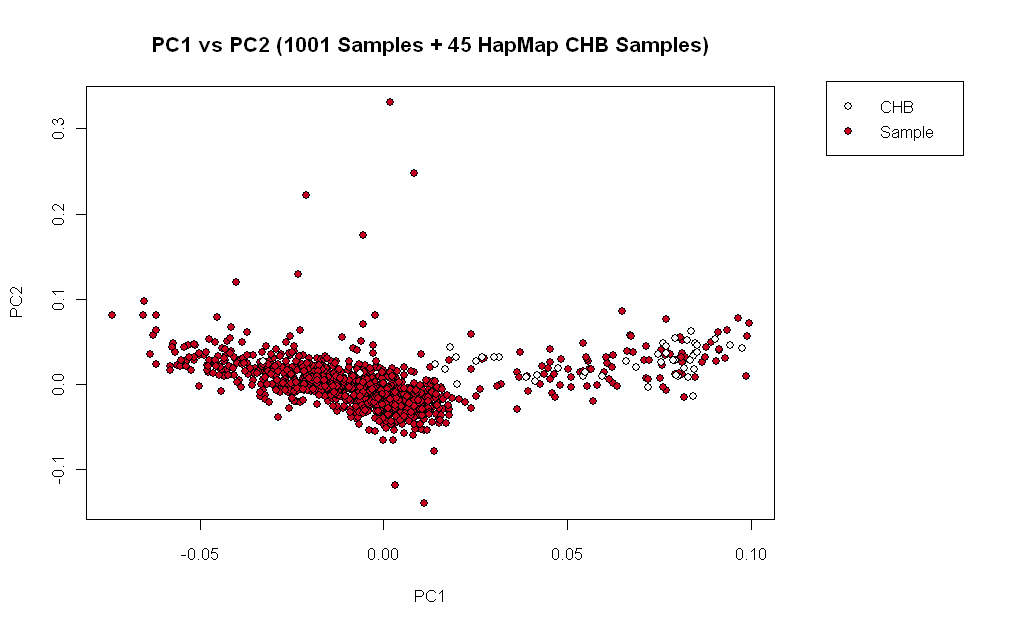


##

## Supplementary Table S1: Concordance correlation coefficients for r^2^ values estimated between Singapore Chinese and other Hapmap populations for SNPs on Chromosome 5.

| **Hapmap Population** | **Correlation** |
| --- | --- |
| **CHB** | 0.89 |
| **JPT** | 0.75 |
| **CEPH** | 0.62 |
| **YRI** | 0.36 |
